# Therapeutic effects of diclofenac, pregabalin, and duloxetine on disuse-induced chronic musculoskeletal pain in rats

**DOI:** 10.1038/s41598-018-21429-3

**Published:** 2018-02-19

**Authors:** Yusuke Ohmichi, Mika Ohmichi, Nobuhito Murai, Masaya Yasui, Nobuaki Takeshita, Hidehiro Oshibuchi, Munekazu Naito, Takashi Nakano, Jun Sato

**Affiliations:** 1 Department of Anatomy, Aichi Medical University, 1-1 Yazakokarimta, Nagakute, Aichi 480-1195 Japan; 2 Pharmacology Research Laboratories, Drug Discovery Research, Astellas Pharma Inc., 21, Miyukigaoka, Tsukuba-shi, Ibaraki, 305-8585 Japan; 3 Department of Psychiatry, Tokyo Women’s Medical University, Kawada-cho 8-1, Shinjuku-ku, Tokyo, 162-8666 Japan; 4 Research Center for Next-Generation Drug Development, Research Institute of Environmental Medicine, Nagoya University, Nagoya, 464-8601 Japan

## Abstract

The aim of this study was to clarify the mechanism of disuse-induced muscle hyperalgesia through the evaluation of the pharmacological behaviour of muscle hyperalgesia profiles in chronic post-cast pain (CPCP) rats with acute and chronic-phase mirror-image muscle hyperalgesia treated with diclofenac (NSAID), pregabalin (an inhibitor of Ca^2+^ channel α2δ), and duloxetine (SNRI). After 2 weeks of cast immobilization, the peak cross-sectional area and muscle wet weight of the ipsilateral soleus and gastrocnemius muscles decreased more significantly in CPCP rats than in untreated rats. Histological findings revealed disuse-induced muscle atrophy in CPCP rats. The blood biochemical parameters of CPCP rats in acute and chronic phases did not differ significantly from those of untreated rats. The diclofenac and pregabalin-treated groups exhibited no improvement in acute or chronic muscle hyperalgesia. In contrast, the duloxetine-treated group exhibited an improvement in acute muscle hyperalgesia, but showed no apparent effect on chronic muscle hyperalgesia on ipsilateral or contralateral sides. However, the chronic muscle hyperalgesia was reversed by intrathecal administration of DAMGO (a μ-opioid receptor agonist). The results suggest that chronic muscle hyperalgesia in CPCP rats did not result from an inflammatory mechanism, and there is only a low probability that it’s caused by a neuropathic mechanism.

## Introduction

Kinesiophobia is the fear of movement and physical activity, which is (wrongfully) assumed to cause (re)injury^1^. According to Vlaeyen and Linton^2^, it evolves when pain, possibly caused by injury, is catastrophized and interpreted as being threatening, leading to avoidance behaviours and hypervigilance to bodily sensations^2^. Avoidance behaviours result in disability, disuse, and depression, which, in turn, lead to the persistence of the pain experience, thereby fuelling the vicious circle of increasing fear and avoidance^2^. Physical disuse leads to physical changes^3–5^, such as muscle and bone atrophy, obesity, and changes in muscle fibre composition, as well as various functional problems, such as reduced cardiovascular function, muscle strength, and motor control. It also results in mental and psychological issues, such as mental anguish, anxiety, and depression, as well as social issues, such as limitation of social activity and economic loss. This whole process is called ‘disuse syndrome’^6^.

Complex regional pain syndrome type I (CRPS I), a representative form of intractable chronic pain, exhibits clinical symptoms of chronic spread of various forms of pain, including chronic muscle hyperalgesia, and other sensory abnormalities to regions beyond the site of injury^7–9^. Interestingly, approximately 50% of patients with CRPS receive treatments—such as splinting or cast immobilization—where the musculoskeletal system enters into a state of disuse^10–12^ A study on disuse of healthy arms due to immobilization (cast) reported cold and mechanical hyperalgesia in the immobile limb^13^. We observed similar results in an experiment involving healthy rats with immobilized hind limbs^14^. After cast immobilization of one hind limb for 2 weeks, we found chronic muscle hyperalgesia expanding not only to the immobilized side, but also to the contralateral side in the calf muscle area^15^. We then found that changes in spinal plasticity were responsible for the mirror image muscle hyperalgesia^15^. Considering these facts, it can be claimed that disuse due to physical immobilization or avoidance of physical movement because of pain could be an essential factor in pain becoming pathogenic.

Inhibitors of the Ca^2+^ channel α2δ, nonsteroidal anti-inflammatory drugs (NSAIDs), and serotonin-norepinephrine reuptake inhibitors (SNRIs) are first-choice drugs prescribed for the treatment of pain caused by injury, inflammation, neuropathy, and similar conditions. However, the mechanism by which disuse induces pain or causes pain to become chronic remains poorly understood, and a definitive treatment for this condition has yet to be developed. Studies on drug therapy for patients with CRPS I, many of whom had disuse-induced CRPS^10–12^, have failed to demonstrate the efficacy of NSAIDs such as diclofenac; these drugs are regarded as having little therapeutic value^16,17^. The inflammation-like symptoms observed in CRPS I and general inflammation associated with trauma or the like are believed to have different aetiologies. Neurogenic inflammation has attracted attention as a possible mechanism of onset of CRPS^16,18,19^. Neuroleptics (e.g. gabapentin and pregabalin)^20,21^, which are among the drugs of first choice for neuropathic pain, have been reported to have some effect on CRPS I^16,22^. While CRPS I is classified as chronic pain not caused by apparent neuropathy^23^, it seems that such clinical results implicate complex neuropathy as one of the causes of pain in CRPS I.

In addition to neuroleptics, SNRIs (such as venlafaxine, milnacipran, and duloxetine) are also recommended as drugs of first choice for neuropathic pain^21^. Assuming that part of the mechanism of onset of pathogenic pain in CRPS overlaps with that of neuropathic pain, it can be expected that SNRIs would be effective against CRPS. However, to our knowledge, no study has demonstrated the efficacy of SNRIs in CRPS. They have, however, been demonstrated to be effective in fibromyalgia^24^, which involves extensive chronic muscle hyperalgesia similar to that in CRPS, and in low-back pain^25,26^, which—similarly to CRPS—involves complex causes of pain^20,27^. These results strongly suggest that pain is caused by transformations in the central nervous system (such as reduced functioning of the descending-pain inhibitory system) as well as psychosocial factors^24,25,28^. Nevertheless, for chronic low-back pain, SNRIs, too, have been reported to be poorly effective^29^, which is indicative of the challenges in clinical research. A major barrier in clinical investigation is the fact that, as observed in CRPS and low-back pain, pain and its parallel symptoms (e.g. numbness, oedema, and abnormal skin-colour changes) change from the acute to the chronic phase over the course of time^18,19^. As has been stated in many reports, the chronic phase especially involves plastic changes in the central nervous system^18,19,30^; consequently, phase changes in pain are also expected to cause changes in the mechanism of pain induction.

A potentially effective method for resolving these problems is investigation using an animal model that has a single origin of pain and allows a clear definition of the timing of changes in pain. In our chronic post-cast pain (CPCP) model^14^, rats have one hind limb immobilized by a cast, which acts as the origin of pain. Pain can be categorized as acute pain, where peripheral dysfunction and pain exist in parallel, or chronic pain, where pain continues to intensify and spread even after recovery from the dysfunction^14^. The spread of pain in CPCP rats is similar to the spread of muscle hyperalgesia to the unaffected side reported in patients with various forms of chronic pain^31^. Investigation with CPCP rats—which experience disuse-induced pain and are largely reflective of the clinical symptoms of patients—is very useful for elucidating the mechanism(s) of onset of disuse-induced muscle hyperalgesia at different stages. The purpose of the present study was to evaluate the effects of diclofenac (an NSAID), pregabalin (an inhibitor of the Ca^2+^ channel α2δ), and duloxetine (an SNRI)—on acute and chronic-phase muscle hyperalgesia in CPCP rats in order to verify the pharmacological behaviour of muscle hyperalgesia profiles in these rat models.

## Results

### Disuse muscle atrophy induced by 2 weeks of cast immobilization

**Figure 1:**
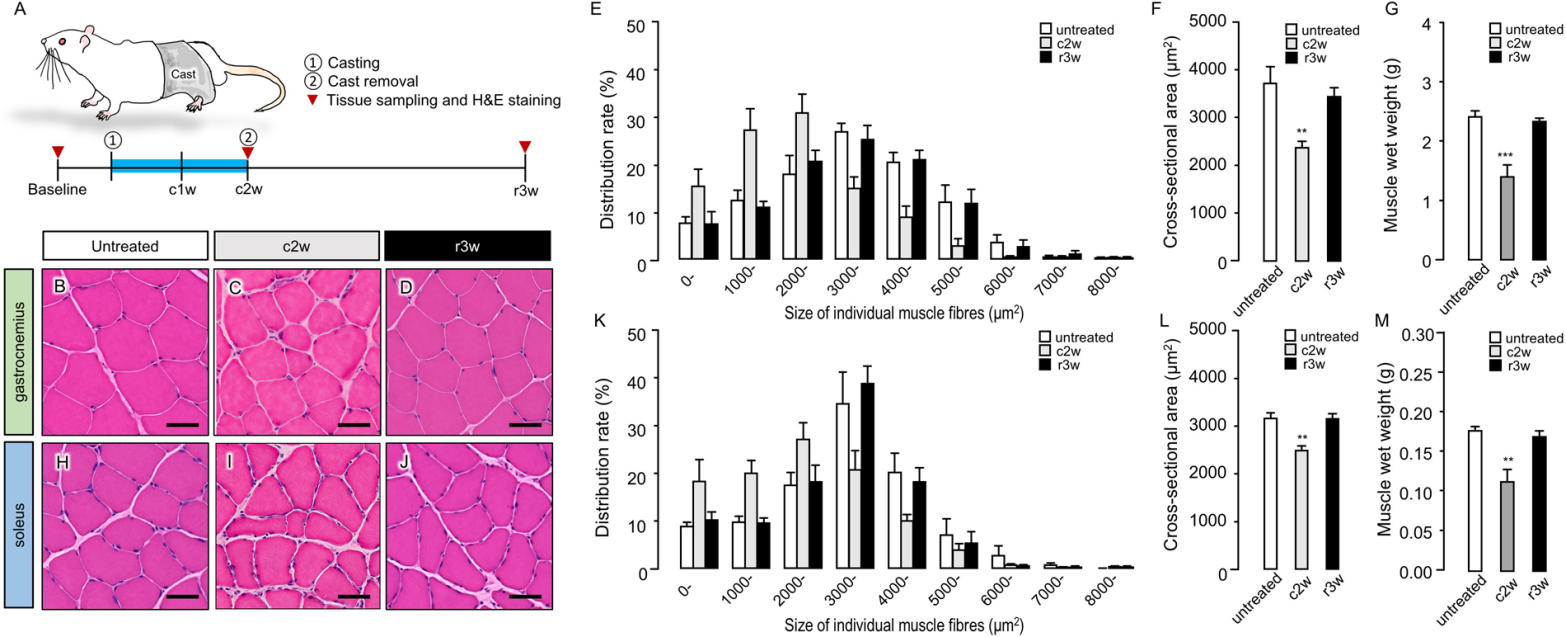
Disuse muscle atrophy induced by 2 weeks of cast immobilization. (**A**) Illustration of a chronic post-cast pain rat model during cast immobilization. Schematic diagram of the experimental protocol for B-D and H-J. Top row (**B–G**): gastrocnemius muscle (GM). Bottom row (**H–M)**: soleus muscle (SM). The first (**B**,**H**), second (**C,U**), and third (**D,J**) columns, which present the results of haematoxylin and eosin staining of muscle tissue specimens of untreated rats (baseline), chronic post-cast pain (CPCP) rats at the end of 2 weeks after cast application (c2w), and CPCP rats at 3 weeks after cast removal (r3w), reveal the differences in size of individual muscle fibres between baseline, c2w, and r3w. The histological findings were confirmed by quantitative analysis (**E–G,K–M**) by comparison between untreated rats (n = 6), CPCP rats at c2w (n = 6), and r3w (n = 5). (**E,K**) Size distribution of individual GM and SM fibres, respectively, baseline, c2w, and r3w. (**F,L**) Results of comparison of average size of individual GM and SM fibres, respectively, between baseline, c2w, and r3w. (**G,M**) Results of comparison of GM and SM wet weight, respectively, between baseline, c2w, and r3w. Data are presented as mean values ± standard error of the mean. Scale bar, 50 µm. ^**^P < 0.01, and ^***^P < 0.001 relative to the untreated group (one-way ANOVA with Dunnett’s multiple comparison test).

In untreated rats, the peak cross-sectional area of the gastrocnemius muscle (GM) (Fig. 1B) ranged from 3000 to 4000 µm^2^ (Fig. 1E), while the mean cross-sectional area and muscle wet weight of the GM were 3448 ± 152.2 µm^2^ (Fig. 1F) and 2.41 ± 0.096 g (Fig. 1G), respectively. After 2 weeks of cast fixation, the peak cross-sectional area of the GM (Fig. 1B) ranged from 2000 to 3000 µm^2^ (Fig. 1E); the mean cross-sectional area (2281 ± 114.7 µm^2^; Fig. 1F, P = 0.0034) and muscle wet weight (1.40 ± 0.202 g; Fig. 1G, P = 0.0029) were significantly decreased as compared to the corresponding baseline values. Three weeks after cast removal, the peak cross-sectional area of the GM (Fig. 1D) ranged from 3000 to 4000 µm^2^ (Fig. 1E); the mean cross-sectional area (3440 ± 193.6 µm^2^; Fig. 1F, P = 0.6962) and muscle wet weight (2.32 ± 0.058 g; Fig. 1G, P = 0.9123) also recovered to baseline values. The peak cross-sectional area of the soleus muscle (SM) (Fig. 1H) in untreated rats ranged from 3000 to 4000 µm^2^ (Fig. 1K), while the mean cross-sectional area and muscle wet weight of the SM were 3150 ± 122.0 µm^2^ (Figs. 1L) and 0.18 ± 0.005 g (Fig. 1M), respectively. After 2 weeks of cast fixation, the peak cross-sectional area of the SM (Fig. 1I) ranged from 2000 to 3000 µm^2^ (Fig. 1K), the mean cross-sectional area (2475 ± 100.2 µm^2^; Fig. 1L, P = 0.0038) and muscle wet weight (0.11 ± 0.016 g; Fig. 1M, P = 0.0029) were significantly decreased as compared to the corresponding baseline values. Three weeks after cast removal, the peak cross-sectional area of the SM (Fig. 1J) ranged from 3000 to 4000 µm^2^ (Fig. 1K); the mean cross-sectional area (3139 ± 110.3 µm^2^; Fig. 1L, P = 0.9978) and muscle wet weight (0.17 ± 0.007 g; Fig. 1M, P = 0.8976) recovered to baseline values. On the basis of these results, the 2-week cast immobilization process was considered to have caused disuse muscle atrophy.

### Long-lasting bilateral muscle hyperalgesia after recovery of disuse-induced physical changes in CPCP rats

**Figure 2:**
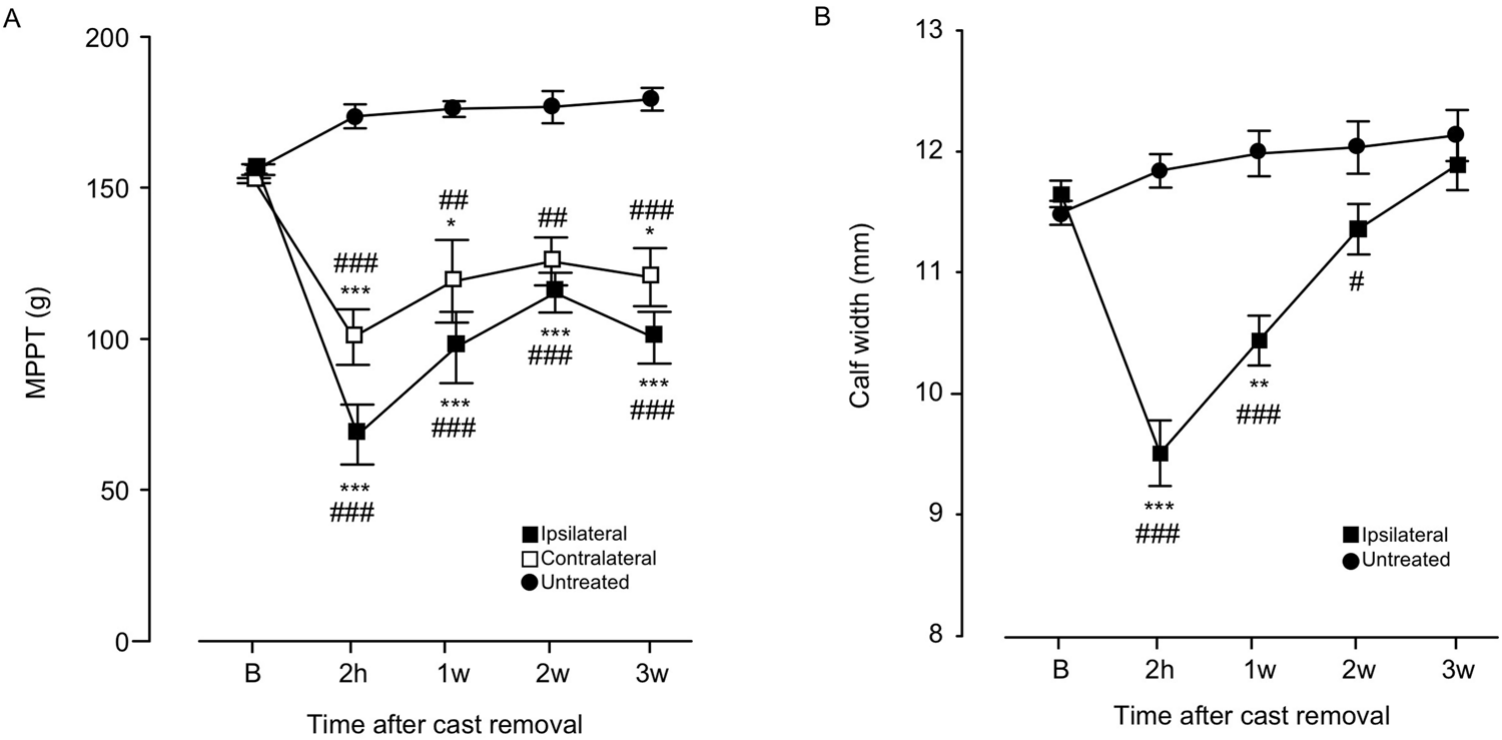
Time-course changes in calf muscle pressure pain threshold (MPPT) (**A**) and width (**B**) after cast removal. Data are presented as mean values ± standard error of the mean (n = 10). Horizontal axes indicate measurement time points (B: before cast application; 2 h: 2 hours; w- weeks). ^*^P < 0.05 and ^**^P < 0.01 relative to B (two-way ANOVA with Dunnett’s multiple comparison test). ^#^P < 0.05, ^##^P < 0.01, and ^###^P < 0.001 relative to the untreated group [**A**] one-way ANOVA with Dunnett’s multiple comparison test, [**B**] Student’s *t*-test).

Chronic post-cast pain was induced by 2 weeks of cast immobilization of the left hind limb. Although the muscle pressure pain threshold (MPPT) decreased bilaterally after cast removal (ipsilateral: F(4, 20) = 8.81840, P = 0.0003; contralateral: F(4, 20) = 7.87558, P = 0.0006), it lasted for 3 weeks (Fig. 2A). In contrast, the calf width (an indicator of muscle atrophy) recovered to the baseline level within 3 weeks of cast removal (Fig. 2B), which is consistent with our previous findings^14^. Therefore, in the present study, we defined the acute phase as 1 week after cast removal, when there was lingering muscle atrophy, and chronic phase as 3 weeks after cast removal, when the significant decrease in MPPT still persisted.

### Changes in blood chemical parameters in CPCP rats

Compared to the untreated control group values, there were no significant changes in the serum concentrations of electrolytes (Na, K, or Cl), enzymatic markers of muscle injury (LDH or CPK), or inflammatory marker (CRP) in CPCP rats after 2 weeks of cast immobilization and at 1 or 3 weeks after cast removal (Table 1). However, there were significant changes in the enzymatic markers of muscle injury 2 hours after ischemic reperfusion (IR) in the hind paw (Table 1), as compared to the control group values. No significant changes were observed in the levels of electrolytes and inflammatory markers in IR rats (Table 1).

**Table 1:** Serum sodium (Na), potassium (K), chloride (Cl), lactate dehydrogenase (LDH), creatine phosphokinase (CPK), and C-reactive protein (CRP) concentrations 2 hours, 1 and 3 weeks after cast removal, and 2 hours after ischemic reperfusion (IR).

	Untreated	2-hour	1-week	3-week	IR-2-hour
LDH (U/L)	69.83 ± 6.42	69.83 ± 6.42 n.s.	66.20 ± 5.65 n.s.	74.20 ± 6.45 n.s.	1375.60 ± 564.9^*,#^
CPK (U/L)	90.33 ± 7.39	152.60 ± 8.67 n.s	104.00 ± 6.15 n.s.	100.4 ± 8.21 n.s	3363.80 ± 992.14^***,##^
Na (mEq/L)	141.17 ± 0.40	143.60 ± 1.29 n.s	140.20 ± 0.37 n.s.	140.80 ± 0.86 n.s.	141.60 ± 1.08n.s.
Cl (mEq/L)	99.00 ± 0.63	102.20 ± 0.86 n.s.	100.20 ± 0.37n.s.	99.20 ± 0.58 n.s.	100.60 ± 0.68 n.s.
K (mEq/L)	4.983 ± 0.183	4.760 ± 0.103n.s.	4.980 ± 0.248 n.s.	5.720 ± 0.302 n.s.	4.940 ± 0.201 n.s.
CRP (mg/dL)	<0.05	<0.05 n.s.	<0.05 n.s.	<0.05 n.s.	<0.05 n.s.

Values are presented as mean ± standard error of the mean (n = 5). n.s.: no significant, *P < 0.05 and ***P < 0.001 difference relative to the untreated group (Scheffe’s multiple comparison test). ^#^P < 0.05 and ^##^P < 0.01 relative to the group of 2 hours after cast removal.

### Effects of diclofenac administration on bilateral muscle hyperalgesia in CPCP rats

**Figure 3:**
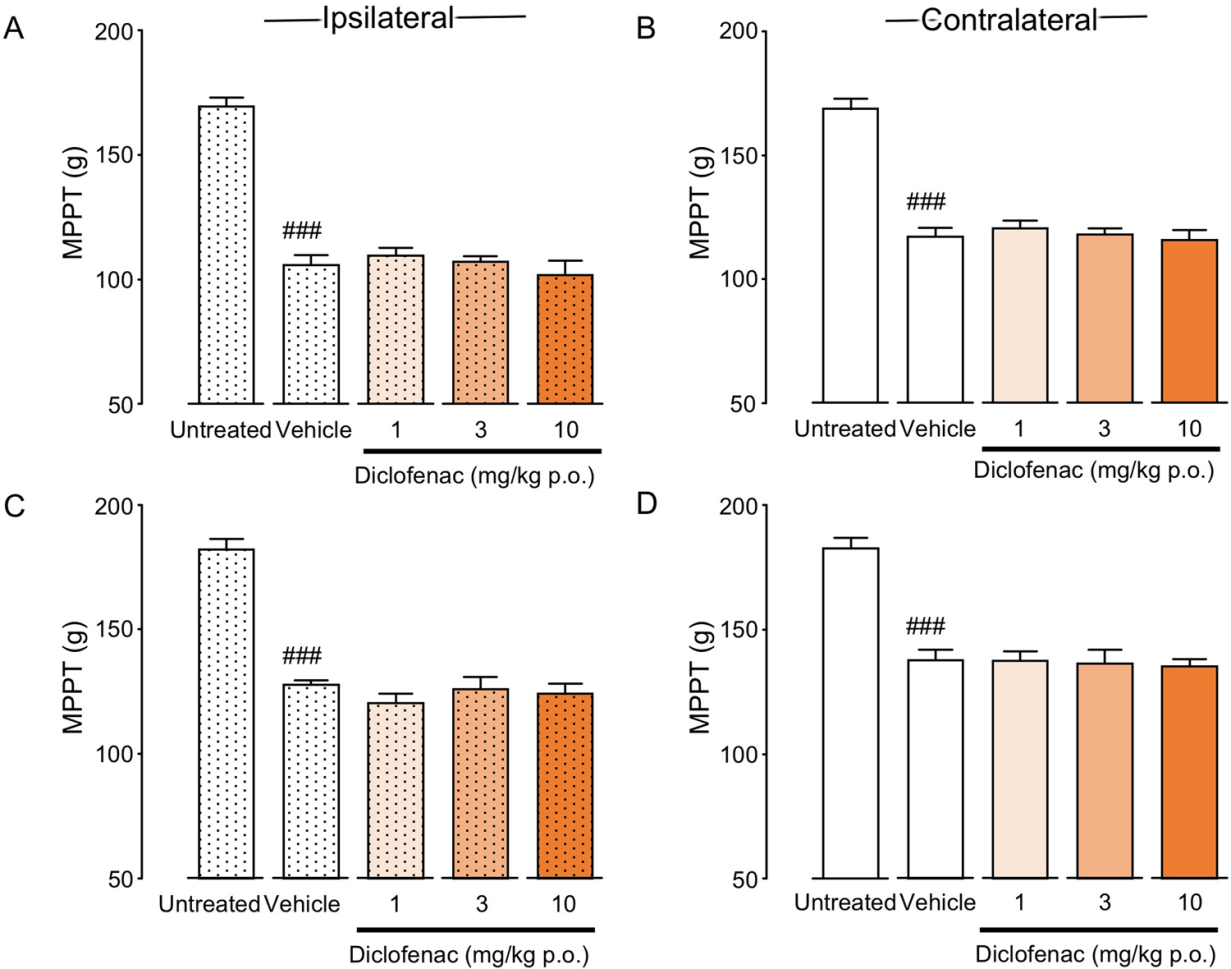
Analgesic effects of diclofenac on muscle pressure pain threshold (MPPT) in chronic post-cast pain rats (**A–D**). Diclofenac was orally administered before the muscle pressure test at 1 (**A**,**B**) and 3 (**C**,**D**) weeks after cast removal. Bars represent mean values ± standard error of the mean (n = 5–6). ^###^P < 0.001 relative to the untreated group (Student’s *t*-test).

Single-dose oral administration of diclofenac was initiated 1 or 3 weeks after cast removal. There were no significant differences in bilateral MPPT between the vehicle and diclofenac-treated groups at 1 (ipsilateral: F(3, 18) = 0.619, P = 0.6119; contralateral: F(3, 18) = 0.313, P = 0.8159) or 3 weeks after cast removal (ipsilateral: F(3, 16) = 0.649, P = 0.5949; contralateral: F(3, 16) = 0.078, P = 0.9708) (Fig. 3A–D).

### Effects of pregabalin administration on bilateral muscle hyperalgesia in CPCP rats

**Figure 4:**
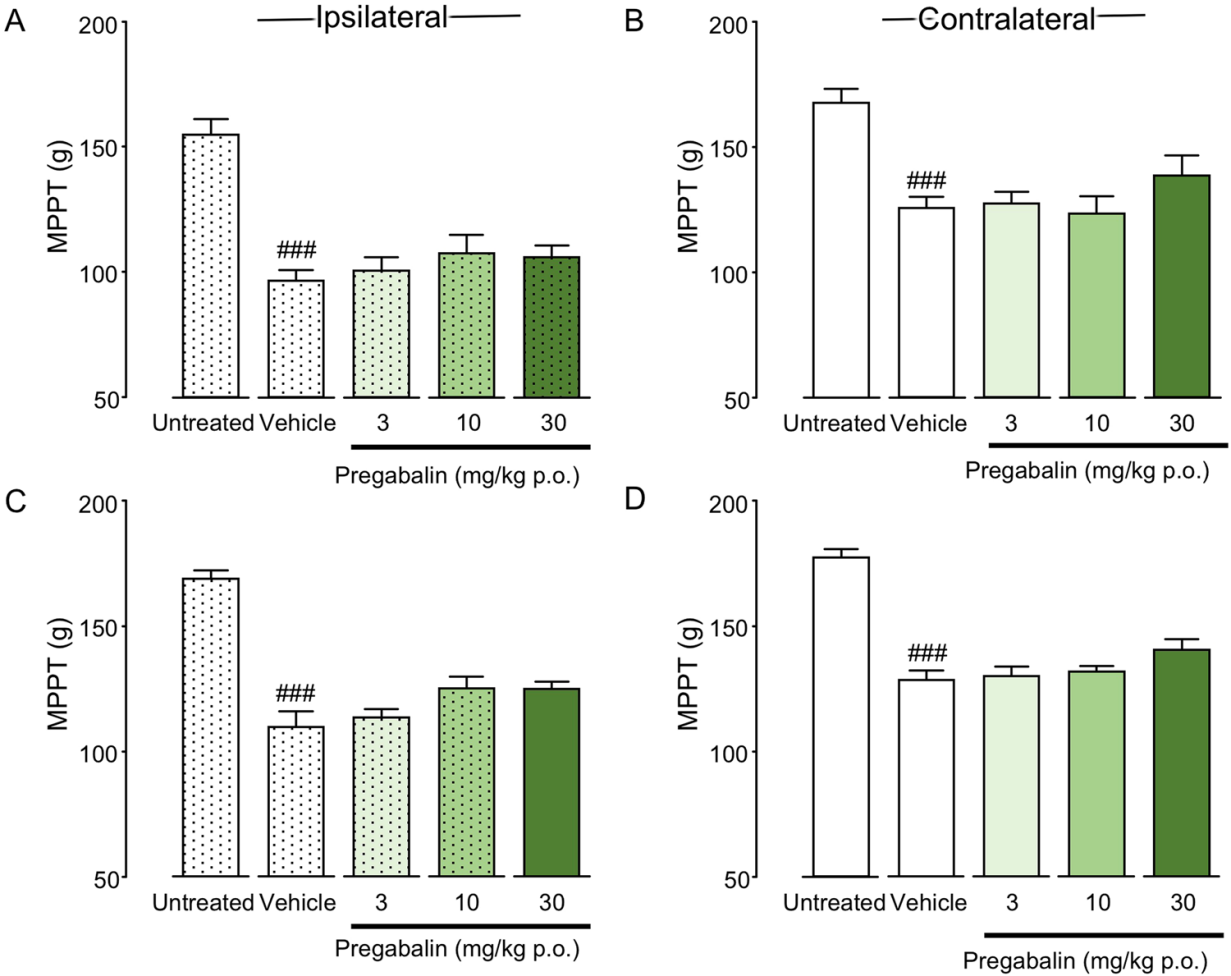
Analgesic effects of pregabalin on muscle pressure pain threshold (MPPT) in chronic post-cast pain rats (**A–D**). Pregabalin was orally administered before the muscle pressure test at 1 (**A**,**B**) and 3 (**C**,**D**) weeks after cast removal. Bars represent mean values ± standard error of the mean (n = 5–6). ^###^P < 0.01 relative to the untreated group (Student’s *t*-test).

Single-dose oral administration of pregabalin was initiated 1 or 3 weeks after cast removal. There were no significant differences in bilateral MPPT between the vehicle and pregabalin-treated groups at 1 (ipsilateral: F(3, 19) = 0.852, P = 0.4827; contralateral: F(3, 19) = 1.136, P = 0.3602) or 3 weeks after cast removal (ipsilateral: F(3, 16) = 3.062, P = 0.0583; contralateral: F(3, 16) = 2.208, P = 0.1268) (Fig. 4A–D).

### Effects of duloxetine administration on bilateral muscle hyperalgesia in CPCP rats

**Figure 5:**
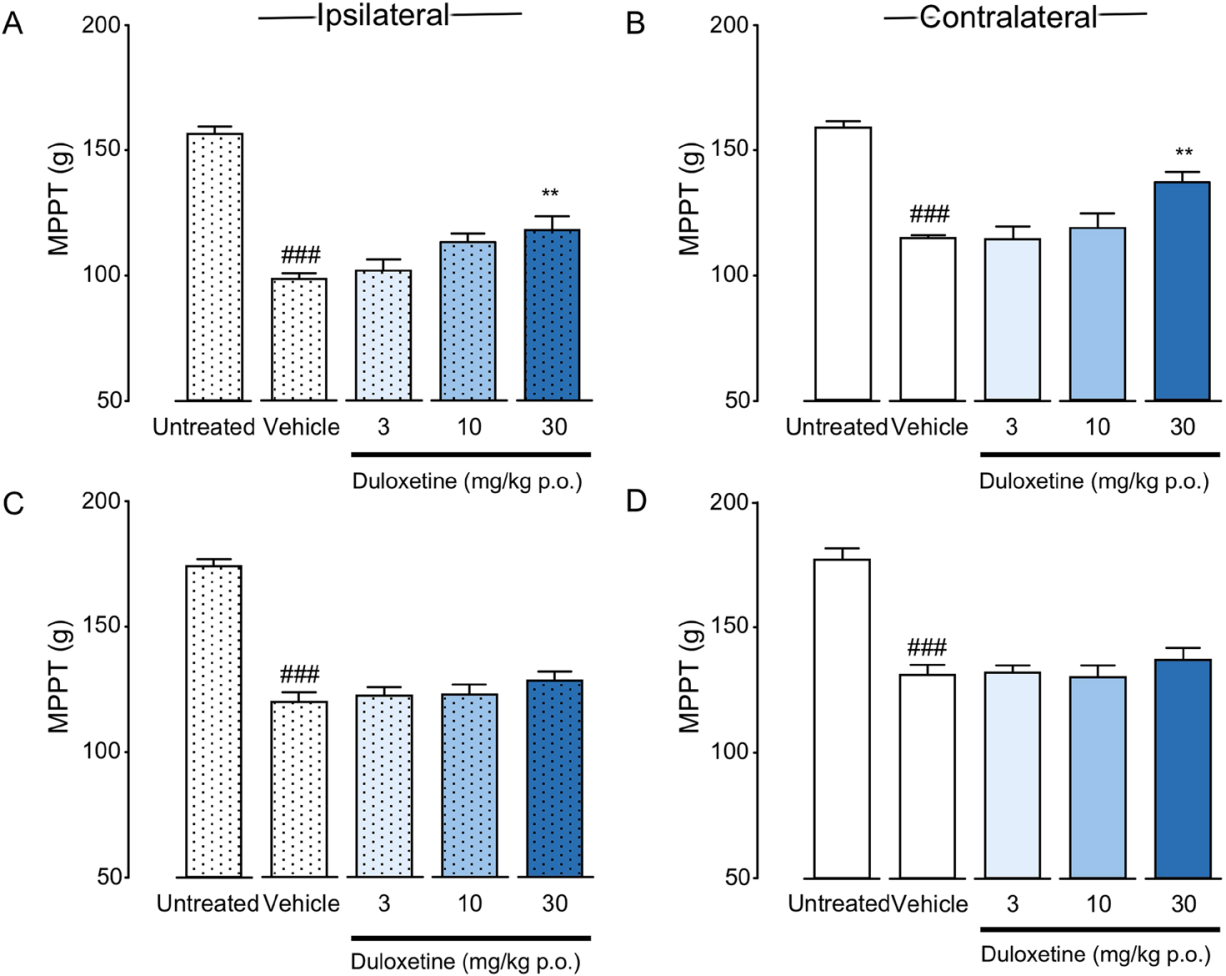
Analgesic effects of duloxetine on muscle pressure pain threshold (MPPT) in chronic post-cast pain rats. Duloxetine was orally administered before the muscle pressure test at 1 (**A**,**B**) and 3 (**C**,**D**) weeks after cast removal. Bars represent mean values ± standard error of the mean (n = 5–6). ^###^P < 0.001 relative to the untreated group (Student’s *t*-test). ^**^P < 0.01 relative to the vehicle-treated group (one-way ANOVA with Dunnett’s multiple comparison test).

Single-dose oral administration of duloxetine was initiated 1 or 3 weeks after cast removal. Duloxetine administration at 1 week after cast removal induced significant recovery in MPPT bilaterally (ipsilateral: F(3, 19) = 4.831, P = 0.0115; contralateral: F(3, 19) = 5.661, P = 0.0061) (Fig. 5A,B). However, duloxetine administration at 3 weeks after cast removal resulted in no significant differences in bilateral MPPT between the vehicle and duloxetine-treated groups (ipsilateral: F(3, 16) = 0.908, P = 0.4592; contralateral: F(3, 16) = 0.578, P = 0.6381) (Fig. 5C,D).

### Effects of repeated duloxetine administration on bilateral muscle hyperalgesia in CPCP rats

**Figure 6:**
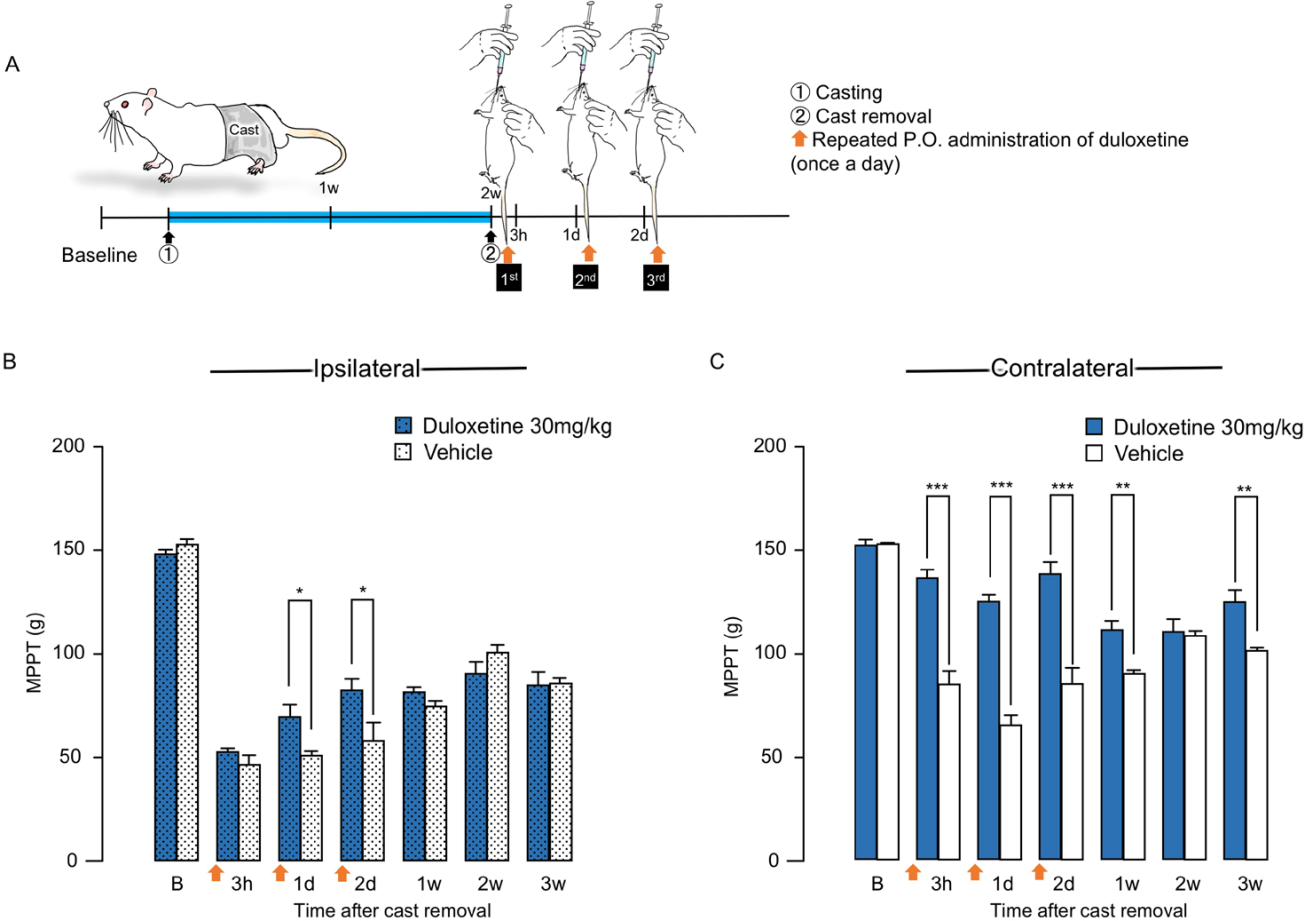
Analgesic effects of repeated administration of duloxetine on muscle pressure pain threshold (MPPT) in chronic post-cast pain rats. (**A**) Schematic illustration of experimental protocol for (**B**,**C)**. Duloxetine was orally administered before the muscle pressure test at 3 hours, 1 day, and 2 days after cast removal. Time-course changes in ipsilateral calf MPPT (**B**) and contralateral calf MPPT (**C**) after cast removal. Bars represent mean values ± standard error of the mean (n = 6). *P < 0.05, **P < 0.01, and ***P < 0.001 relative to the vehicle-treated group (Student’s t-test).

For this experiment, duloxetine (30 mg/kg) administration (once daily for three days) was initiated just after cast removal (Fig. 6A). It induced significant recovery in ipsilateral MPPT at 1 day and 2 days after cast removal (F(6, 30) = 37.3983, P < 0.0001) (Fig. 6B). However, there were no significant differences in ipsilateral MPPT after third administration, 1 week after cast removal (Fig. 6B). Duloxetine induced significant recovery in contralateral MPPT at 3 hours, 1 day, 2 days, 1 week, and 3 weeks after cast removal (F(6, 30) = 11.1240, P < 0.0001) (Fig. 6C).

### Effects of DAMGO administration on bilateral muscle hyperalgesia in CPCP rats

**Figure 7:**
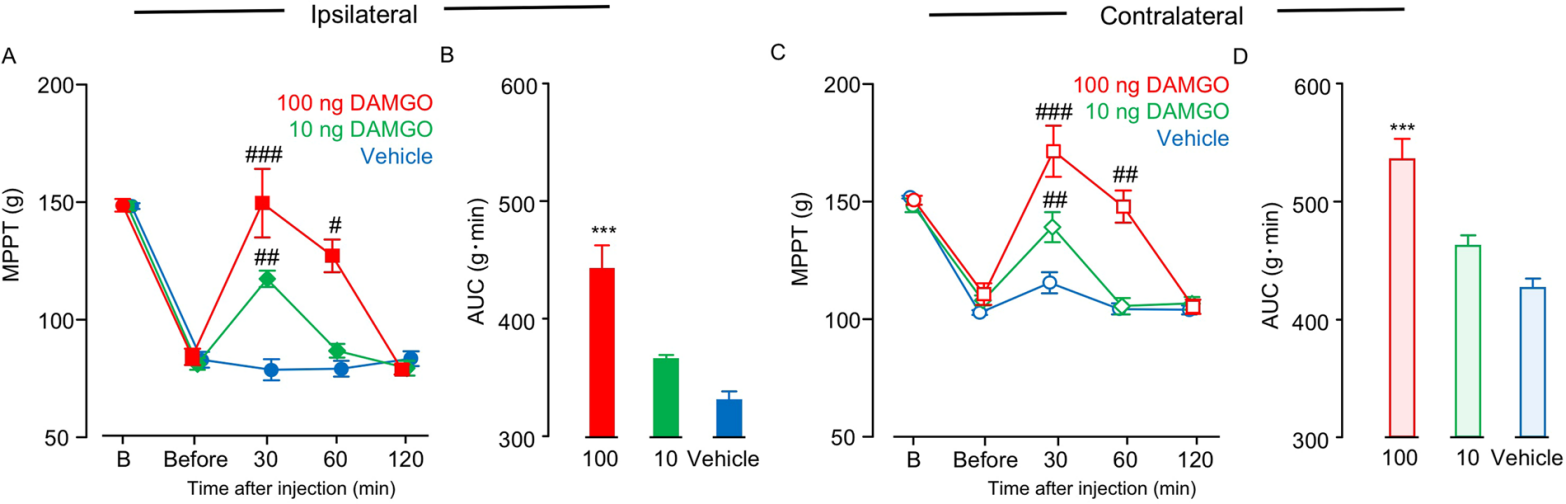
Analgesic effects of intrathecal administration of DAMGO on muscle pressure pain threshold (MPPT) in chronic post-cast pain rats. Time-course changes in ipsilateral calf MPPT (**A**) and contralateral calf MPPT (**C**) after DAMGO administration. Horizontal axes indicate measurement time points (B: before cast application; Before: before DAMGO administration). (**B**) and (**D**) represent the area under the curve for (**A**) and (**C**). Bars represent mean values ± standard error of the mean (n = 6). ^#^P < 0.05, ^##^P < 0.01, and ^###^P < 0.001 relative to B (two-way ANOVA with Dunnett’s multiple comparison test). ***P < 0.001 relative to the vehicle-treated group (one-way ANOVA with Dunnett’s multiple comparison test).

We evaluated dose-dependent efficacy of single-dose intrathecal administration of DAMGO on the bilateral MPPT 3 weeks after cast removal (Fig. 7). Bilateral MPPT increased significantly in a dose-dependent manner 30 min after the DAMGO administration (ipsilateral: F(2, 12) = 18.844, P = 0.0001; contralateral: F(2, 12) = 20.077, P = 0.0002) (Fig. 7A–D). However, the effect of DAMGO on bilateral MPPT disappeared 2 hours after the i.t. injection (Fig. 7A,C).

## Discussion

In pain treatment, NSAIDs are generally used for inflammatory pain, while neuroleptics and SNRIs are used as first-line drugs for neuropathic pain. Validation of efficacies of these three types of painkillers is important for elucidating the mechanism of mechanical muscle hyperalgesia caused by physical disuse. The results of our study validate the efficacies of diclofenac (an NSAID), pregabalin (an inhibitor of the Ca^2+^ channel α2δ), and duloxetine (an SNRI) in treatment of acute and chronic-phase mirror-image muscle hyperalgesia in CPCP rats. Of the three tested drugs, only duloxetine exhibited an analgesic effect on muscle pain bilaterally, in the acute phase of muscle hyperalgesia, 1 week after cast removal (Fig. 5A,B). In the chronic phase of muscle hyperalgesia, none of the drugs were effective for treatment of muscle hyperalgesia bilaterally (Figs 3–5C,D). The results of the drug tests suggest a high probability that chronic muscle hyperalgesia caused by disuse is a new pathology different from inflammatory and neuropathic pain.

Diclofenac had no effect on acute or chronic-phase muscle hyperalgesia in CPCP rats (Fig. 3). The results of blood biochemical analysis did not provide any clear evidence of cell damage or inflammation (Table 1), which suggests that muscle pain in this model is unlikely to have been caused by cyclooxygenase-induced general inflammation. Similar to the findings in CPCP rats, NSAIDs have also been demonstrated to be ineffective in patients with CRPS I^16^, since neurogenic inflammation has been noted as being an important cause of pain in CRPS I^18,19,30^. Two previous studies^32,33^ compared the effects of immobilization in two groups of rats—a fracture group, comprising rats with immobilization after fracture, and a sham group, comprising healthy rats with immobilization but no fracture of limbs. After 4 weeks of cast immobilization, both groups exhibited neurogenic inflammation, which suggested that the inflammation was caused by immobilization alone, regardless of fracture^32,33^. Thus, neurogenic inflammation is highly likely to be essential to the induction of disuse-related pain.

Pregabalin, like diclofenac, also exhibited only occasional effects on muscle hyperalgesia in CPCP rats (Fig. 4). Therefore, it seems unlikely that peripheral neuropathy is the cause of muscle hyperalgesia in CPCP rat models. Pregabalin, as a drug of first choice for neuropathic pain, reportedly has an analgesic effect in clinical settings^21,34–36^. It has also been reported to exhibit a high analgesic effect in representative neuropathic pain models, including spinal^37,38^ and partial^39^ nerve ligation and chronic constriction injury^38,40^ models. As previously described, under the assumption that cast immobilization possibly induces neuropathic pain by nerve compression, on the last day of cast immobilization, we tried to verify whether an immune response is associated with activating transcription factor 3 (ATF3)—a neuronal marker of nerve injury—in the dorsal root ganglion (DRG) neurons of L3–5, which dominate the immobilized part^15^. We confirmed that none of the DRG neurons exhibited a significant increase in ATF3 immune response, which led us to conclude that cast immobilization does not directly cause nerve injury^15^. The fact that pregabalin was not found to be effective against muscle hyperalgesia in CPCP rats in the present study provides pharmacological support to the notion that muscle hyperalgesia in this model is not caused by neuropathy.

Serotonin-norepinephrine reuptake inhibitors, such as duloxetine, are thought to provide analgesia by inhibiting serotonin (5-HT) and noradrenaline (NA) reuptake, thereby promoting the excitement of NA and serotonin neurons descending through the anterior funiculus and suppressing the activity of nociceptive neurons in the dorsal horn of the spinal cord^41^. Thus, the reduction in acute-phase muscle hyperalgesia observed in CPCP rats (Fig. 5A,B) presumably arises from the effect of descending-pain inhibition on the activity of nociceptive neurons in the immobilized limb^42,43^. However, because no analgesic effect was observed on either side in the chronic phase (Fig. 5C,D), and acute-phase muscle hyperalgesia on either side could not be fully relieved (Fig. 5A,B), some kind of pathological change in the pain system is inferred to have possibly started already in week 1 after cast removal, which is defined as the acute phase.

To investigate the involvement of descending-pain inhibitory system, we assumed that continuous administration of duloxetine at an earlier stage after cast removal could suppress muscle hyperalgesia in CPCP rats. We evaluated the effect of duloxetine administered immediately after cast removal once daily for three continuous days (Fig. 6). On the immobilized side (Fig. 6B), the muscle hyperalgesia attenuated transiently only during the continuous administration, but after the continuous administration, the muscle hyperalgesia recurred and continued until 3 weeks after cast removal. In a previous study, we found that the muscle hyperalgesia was suppressed by administration of reactive oxygen scavengers (Tempol or NAC) 24 hours after cast removal^14^. We also found an increase in the immune response of pERK in L4 DRG neurons in the immobilized side 24 hours after cast removal^15^. Therefore, we suggest that the onset of muscle hyperalgesia in the immobilized side may be significantly related to a peripheral factor generated in the immobilized hind limb rather than decrease in the function of the descending pain inhibitory system. On the contralateral side (Fig. 6C), the suppression of muscle hyperalgesia continued not only during continuous administration of duloxetine, but also afterward. However, the effect of duloxetine decreased as the time passed after cast removal. Duloxetine administration was less effective in suppression of the chronic muscle hyperalgesia 3 weeks after cast removal. These results suggest that pathological plastic changes in the descending pain inhibitory system could be involved in the expansion of the muscle hyperalgesia to the contralateral side.

We have previously reported that microglia in the dorsal horn of the lumbar spine are activated as early as day 1 after cast removal^14,15^. It has been reported in various animal models that microglial^44,45^ activation in the dorsal horn of the lumbar spine is involved in the induction of pathological pain. Similarly, in CPCP rats, the activation of microglial cells was possibly involved in the induction of disuse-induced muscle hyperalgesia. Yamashita *et al*.^46^ found that duloxetine has an inhibitory effect not only on the reuptake inhibition of 5-HT and NA, but also on the function of the phosphorylating receptor P2X4R, a subtype of ATP-dependent nonselective cation channel in spinal microglia. Tsuda *et al*.^47^ reported that P2X4 (P2X4R) increased the expression of spinal cord microglia after peripheral nerve injury, and blockage of the P2X4R function produces a reversal of mechanical allodynia. Furthermore, it has been demonstrated that disruption of P2X4R gene abolishes mechanical allodynia induced by peripheral nerve injury^48^. Based on these information, we suggest that the activation of microglial cells in the spinal cord was possibly involved in the induction of disuse-induced muscle hyperalgesia in CPCP rat.

Because diclofenac (an NSAID) (Fig. 3C,D), pregabalin (an inhibitor of the Ca^2+^ channel α2δ) (Fig. 4C,D), and duloxetine (an SNRI) (Fig. 5C,D) were ineffective 3 weeks after cast removal (chronic phase), we investigated the effect of opioid analgesia, which is the main choice for severe chronic pain management. We intrathecally (i.t.) administered DAMGO, an agonist of the μ opioid receptor (Fig. 7), which inhibited the bilateral mechanical hyperalgesia in the chronic phase in a concentration-dependent manner. In the immobilized side (Fig. 7A), the DAMGO administration of maximum concentration (100 ng) attenuated MPPT to the baseline level. Interestingly, in the contralateral side (Fig. 7C), DAMGO administration (100 ng) attenuated MPPT, but above the baseline value. Kohno *et al*.^49^ reported that i.t. DAMGO (100 ng) has no effect on basal mechanical sensitivity in naive animals and does not reduce mechanical hypersensitivity in both the spinal nerve ligation (SNL) and spared nerve injury (SNI) models. However, it reduced the mechanical sensitivity produced by peripheral inflammation resulting from intraplantar injection of complete Freund’s adjuvant (CFA). These results showed that sensitivity to opioids in disuse-induced mechanical hypersensitivity in CPCP rats was similar to that observed after inflammation-induced mechanical hypersensitivity, but quite different from that observed after nerve injury. However, diclofenac (an NSAID), which is usually effective for inflammatory pain, was found to be ineffective for mechanical muscle hypersensitivity in CPCP rats (Fig. 3C,D), and there were no changes in inflammatory markers or cytotoxic markers in 3 weeks after cast removal (Table 1). On the basis of these results, disuse-induced muscle hypersensitivity is likely to have a different mechanism from inflammatory pain.

In conclusion, the present study evaluated the pain reduction effects of diclofenac (an NSAID), pregabalin (an inhibitor of the Ca^2+^ channel α2δ), and duloxetine (an SNRI) on mirror-image acute and chronic-phase muscle hyperalgesia in CPCP rats. Of the three drugs, only duloxetine mitigated acute-phase muscle hyperalgesia bilaterally. None of the drugs was effective against chronic-phase muscle hyperalgesia on either side. However, the chronic muscle hyperalgesia was reversed by i.t. administration of an MOR agonist, DAMGO. The results suggest that chronic muscle hyperalgesia in CPCP rats did not result from an inflammatory mechanism, and there is only a low probability that it’s caused by a neuropathic mechanism. However, since anatomical or electrophysiological experiments could not be performed, this possibility cannot be excluded. Further verification of this possibility by the means mentioned would be of interest in future work.

## Methods

### Animals

Experiments in the present study were conducted with the approval of the Animal Care Committee, Aichi Medical University, Aichi, Japan, and the Institutional Animal Care and Use Committee, Astellas Pharma Inc., Tsukuba, Japan, and in accordance with the International Association for the Study of Pain guidelines for pain research in animals. The Tsukuba Research Center of Astellas Pharma Inc. is accredited by the Association of Assessment and Accreditation of Laboratory Animal Care (AAALAC) International. Nine or 10-week-old male Sprague Dawley rats (300–400 g; Japan SLC, Hamamatsu, Japan) were purchased and housed under controlled temperature (23 ± 2 °C) and humidity (55% ± 10%), with a 12-h light/dark cycle. The animals were provided free access to food and water. During the 2-week cast immobilization period, the animals were allowed to move about their cages using both forelimbs and the non-immobilized hind limb, with general activity and food and water intake maintained at levels similar to those before immobilization. For measurement of muscle pressure pain threshold (MPPT) (Fig. 2A) and calf width (Fig. 2B), the rats were restrained with a cloth sock from the head to the pelvis. The animals were habituated to handling and restraint with the sock (1 h daily for 3 days) before the start of experimentation. Once adapted to the process, they typically remained quietly snuggled within the sock, despite being free to emerge; on such occasions, they were gently reintroduced to the restraint. We attest that all efforts were made to minimize the number of animals used and their suffering.

### Hind limb cast immobilization

In accordance with our previous findings, CPCP was induced through a 2-week hind limb cast immobilization procedure^14,15,50^. In brief, a plaster cast (30–35 g; Plasrun-Gyps E; Alcare, Tokyo, Japan) was applied from the pelvis to the middle of the left hind paw (Fig. 1A) under anaesthesia with pentobarbital sodium (64.8 mg/kg, intraperitoneal injection). Rats exhibiting signs of circulation impairment (e.g. congestion, ischemia, or pressure ulcer) in the immobilized hind limb or severe damage to the cast during the 2-week immobilization period were excluded from the behavioural experiments. Animals that completed the 2-week immobilization period were restrained with a comfortable cloth sock, and the casts were removed by hand or, if necessary, scissors. Untreated rats were used as control animals. The following behavioural tests (including those before cast removal) were administered for blinded comparative evaluation.

### Hind paw ischemic reperfusion

A nitrile 70 Durometer O-ring (O-rings West, Seattle, WA) with 7/32 in. internal diameter was placed around the rat’s left hind limb for 3 hours just proximal to the ankle joint under anaesthesia with pentobarbital sodium (64.8 mg/kg, intraperitoneal injection). We standardized the position of the O-ring to a point on the limb just proximal to the medial malleolus^51^. After 3 hours ischemia, the O-rings were removed by scissors.

### Drug administration

The effects of diclofenac, pregabalin, and duloxetine on MPPT were investigated 1 (acute phase) and 3 weeks (chronic phase) after cast removal. Pregabalin and duloxetine were dissolved in distilled water. Diclofenac, purchased from Sigma-Aldrich (St. Louis, MO), was suspended in a 0.5% methylcellulose aqueous solution. The drugs were orally administered (5 mL/kg) at the following dosages (calculated as the free-base form): diclofenac, 1, 3, and 10 mg/kg and pregabalin and duloxetine, 3, 10, and 30 mg/kg. MPPT was measured at 1 h (diclofenac), 2 h (pregabalin), and 3 h (duloxetine) after administration. Each measurement point was selected as the time for demonstrating the maximum plasma concentration^52–54^. Only duloxetine (30 mg/kg) that showed an analgesic effect in the acute phase was evaluated for the effect of continuous administration, three times (once a day), immediately after cast removal. In the chronic phase (3 weeks after cast removal) in which all drugs were ineffective, the effect of i.t. administration of DAMGO, MOR agonist, purchased from Sigma-Aldrich (St. Louis, MO), on bilateral MPPT of CPCP rats was evaluated. DAMGO was dissolved in saline and injected i.t. in a volume of 20 µl/rat by lumber puncture between the spinous processes L4 and L5, 3 weeks after cast removal. The dosage was determined by preliminary experiments (see Supplementary Movies S1, S2).

### Muscle pressure test

Muscle pressure threshold was measured as per a previously described method^14,15,50^. Pressure stimulation of the calf muscle was achieved using a push-pull gauge algometer (Aikoh Engineering, Osaka, Japan). A cone-shaped pusher with a rounded tip (diameter, 2.4 mm) was applied to the belly of the calf muscle with linearly increasing pressure (10 g/s), and the minimum pressure required to elicit foot withdrawal was measured. This process was repeated four times at intervals of at least 30 s, and the median value of the last three trials was defined as the pain threshold. These measurements were also performed on three separate days before cast application, and the average values from these three iterations were used as baseline control values.

### Muscle atrophy

Muscle atrophy was measured as per a previously described method^14,15,50^. For investigating local changes such as calf muscle atrophy in the hind limb, the rats were restrained with a sock from the head to the pelvis and positioned supine. If necessary, the animals were terminally anaesthetized with inhalant isoflurane (2%). Muscle atrophy was evaluated by measuring calf width with manual callipers. The callipers were touched lightly to the thickest area of the calf while holding the hind paw in the maximum extended position. The measurements were repeated three times, and the median of these values was used for further analysis.

### Histological analysis of skeletal muscles

The animals were anaesthetized by intraperitoneal injection of sodium pentobarbital (100 mg/kg), following which, the gastrocnemius muscle (GM) and soleus muscle (SM) were dissected out. The animals were then euthanized by anaesthetic overdose. GM and SM were frozen in liquid nitrogen-cooled isopentane (Wako, Osaka, Japan) and then processed for sectioning on a cryostat. Sections of 10 mm thickness were cut, mounted on slides, and stained with haematoxylin–eosin (Wako, Osaka, Japan). The sections were then dehydrated in a graded series of ethanol (70–100%) and xylene and mounted with the Entellan New Rapid Mounting Medium (Merck, Darmstaldt, Germany). Digital images were acquired with the KeyenceBZ-X710 microscope (Keyence, Osaka, Japan) and analysed using an image-analysis software (Image J 1.15i, National Institutes of Health, Bethesda, MD).

### Evaluation of serum biochemical parameters

Two millilitres of blood was drawn transcardially before casting and at 2 hours, 1 week, and 3 weeks after cast removal, and at 2 hours after ischemic reperfusion in hind paw. The blood was transferred into tubes (#31204, Immuno-Biological Laboratories, Gunma, Japan) and centrifuged at 3000 × g for 10 min at 4 °C to separate serum for analysis. Serum concentrations of sodium (Na), potassium (K), chloride (Cl), lactate dehydrogenase (LDH), creatine phosphokinase (CPK), and C-reactive protein (CRP) were analysed using an automated analyser (Hitachi 7600–11os analyser, Hitachi High-Technologies, Tokyo, Japan), by a specialist who was unaware of the details of this study.

### Statistical analysis

Data are expressed as mean values ± standard error of the mean. Statistical analysis was performed using GraphPad Prism version 5.04 (GraphPad Software, Inc., San Diego, CA) and StatFlex version 6.0 (Artech Co., Ltd., Osaka, Japan). Data were analysed by the chi-square test, Student’s *t*-test, one-way analysis of variance (ANOVA), or two-way ANOVA, followed by Dunnett’s or Scheffe’s multiple comparison test. The datasets analysed during the current study are available from the corresponding author on reasonable request.

### Data availability

No datasets were generated or analysed during the current study.

## Electronic supplementary material

Supplementary Information

Movie S1

Movie S2
