# Supplementary material for: Therapeutic effects of diclofenac, pregabalin, and duloxetine on disuse-induced chronic musculoskeletal pain in rats
Source: Sci Rep. 2018 Feb 19;8:3311. doi: 10.1038/s41598-018-21429-3 (PMC5818528; doi:10.1038/s41598-018-21429-3)
Supplement: Supplementary file 1 — Supplementary Information [file 41598_2018_21429_MOESM1_ESM.pdf]

Supplementary information

Therapeutic effects of diclofenac, pregabalin, and duloxetine on disuse-induced chronic musculoskeletal pain in rats

Yusuke Ohmichi, Mika Ohmichi, Nobuhito Murai, Masaya Yasui, Nobuaki Takeshita, Hidehiro Oshibuchi, Munekazu Naito, Takashi Nakano & Jun Sato

Movie S1. A highly magnified part of the transitional third and fourth lumbar spine vertebrae observed from between the spinous process of Th13 and of L1. Lumbar puncture was performed between L4 and L5; 1% Evans blue dye (EBD) was intrathecally administered and photographed in the medullary cavity through the Th13–L1 intervertebral space. When 10 μl EBD was injected, it did not sufficiently reach to L4 vertebrae of spinal cord. When 20 μl EBD was injected, it sufficiently reached to the L4 spinal vertebrae. Based on this, we selected to use 20 μl of DAMGO by lumbar puncture as a dose in this experiment.

Movie S2. Condition of paralysis of both hind limbs after intrathecal administration of lidocaine by lumbar puncture. 2% Lidocaine (10 μl) or 1% Lidocaine (20 μl) was injected from between the L4-5 intervertebral space. When 10 μl lidocaine was administered, hind paw showed response to pinch stimulation. On the contrary, response to pinch stimulation in the hind paw disappeared when 20 μl lidocaine was administered. This confirmed that 20 μl was sufficient to paralyze the hind paws. Based on the intrathecal administration experiment of Evans blue dye (Movie S1) and the results of this study, the dose of DAMGO by lumbar puncture in this experiment was set to 20 μl.
